# Status of Comorbid Congenital Anomalies and Their Influence on Resource Use in Pediatric Inpatients: A Serial Cross-Sectional Study in Shanghai, China

**DOI:** 10.3389/fpubh.2020.580664

**Published:** 2020-10-30

**Authors:** Jianwei Shi, Ning Chen, Wenya Yu, Rui Liu, Hua Jin, Zhaohu Yu, Li Luo, Li Gu, Rong Yang, Qian Liu, Wei Feng, Zhaoxin Wang

**Affiliations:** ^1^School of Public Health, Shanghai Jiao Tong University School of Medicine, Shanghai, China; ^2^Shanghai General Practice and Community Health Development Research Center, Shanghai, China; ^3^School of Medicine, Tongji University, Shanghai, China; ^4^Shanghai Tenth People's Hospital, Tongji University School of Medicine, Shanghai, China; ^5^Navy 971 Hospital, Qingdao, China; ^6^School of Public Health, Fudan University, Shanghai, China; ^7^Department of Psychological Medicine, Renji Hospital, Shanghai Jiao Tong University School of Medicine, Shanghai, China

**Keywords:** comorbid congenital anomalies, status description, pediatric inpatients, cost, resource

## Abstract

**Objectives:** The status of children with comorbid congenital anomalies (CAs) and their effects on related hospital resource utilization have been minimally investigated. We aimed to describe the congenital anomalies comorbidity status and their effects on hospital resource utilization (length of stay, cost) by pediatric patients.

**Setting:** This study was conducted in five tertiary care children's hospitals in Shanghai, China.

**Participants:** Data were obtained from the inpatients' electronic health records; diagnoses were recorded using International Classification of Disease, Ninth Revision codes. In total, 7,890 children were diagnosed with congenital anomalies (13.13%), which were either primary or secondary.

**Primary and secondary outcome measures:** The dependent variables were length of stay and cost. The independent variables were demographic and clinical characteristics and CA status.

**Results:** In total, 50.98% of the hospitalized patients had comorbid CA conditions. Medical+CA patients were associated with a longer LOS (β = 2.656, *P* < 0.001), and CA+medical patients were associated with higher costs (β = 7.222, *P* < 0.001). Cardiovascular, musculoskeletal, and genitourinary diseases were the top three comorbid diseases. The average LOS for the top three comorbid diseases was longest in the medical+CA group, followed by CA+medical group. Cardiovascular disease was the most frequent comorbidity (ranking 1 in the medical+CA group and 2 in the CA+medical group), and the cost of cardiovascular disease was highest in all groups.

**Conclusions:** A high prevalence of comorbid CA conditions was observed among pediatric inpatients in the sampled tertiary hospitals in China. Strategic planning should be improved to guide resource utilization for complex comorbid CA care.

## Introduction

Due to the tremendous progress in prenatal diagnosis and improvements in health care services brought about by increased focus on primary health care, there has been a significant reduction in infant and childhood mortality rates in most countries over the past two decades (1–4). However, as major causes of infant and childhood mortality, congenital anomalies (CAs) remain a serious problem worldwide (1, 2). Statistics from “Healthy children.org” show that ~3–4% of all infants born in the U.S. had a CAs in 2018 (5). Studies in India indicated that the rate of birth defects and genetic disorders was 2.12% based on samples from three large cities (2005–2007) (6). Data from the WHO from 2018 revealed that CA deaths accounted for 48,977 or 0.54% of total deaths and that the age-adjusted death rate was 4.53 per 100,000 people in China, with a worldwide ranking of 135 (7).

CAs may contribute to long-term disability in children, and hospitalization expenses impose a sizable burden on their families, health systems, and societies (8–10). In recent years, empirical studies in developed countries have begun to focus on the very large hospital resource utilization of patients with CAs. For instance, in the U.S., based on a 20% stratified sample of discharges from nonfederal community hospitals in 2013, Arth et al. (11) estimated the annual cost of birth defect-associated hospitalizations in the U.S. and found that these conditions had disproportionately high costs, accounting for 3.0% of all hospitalizations and 5.2% of total hospital costs. In another U.S. study, based on a sample of 3,407,146 pediatric patients with congenital heart disease (CHD) discharged from 4,121 hospitals, the authors showed that pediatric patients without CHD had a shorter length of stay (LOS) than those with CHD (4.3 vs. 14.4 days) (12). In China, by using the CHD inpatient database (2007–2012) from all secondary and tertiary hospitals in Beijing, China, Cui (13) found that inpatients with severe CHD had a longer LOS and higher average cost than those with mild conditions.

Although the abovementioned studies found that patients with a single CA had markedly improved hospital resource utilization, including hospitalization, LOS and cost (14, 15), the status of comorbid CAs and whether comorbid CAs could significantly affect pediatric hospital resource utilization remains unknown. A comorbidity is considered present with either a primary or secondary diagnosis of a disease (12). Usually, CA comorbidity groups consist of one of the following two types of patients: those with another disease as the primary diagnosis and a CA as the secondary diagnosis and those with a CA as the primary diagnosis and another disease as the secondary diagnosis. Since little is known about how the cooccurrence of a CA and another medical diagnosis differentially influences pediatric hospitalization resource use, the primary aim of this study was to describe the CA comorbidity status and its effects on hospital resource utilization (including LOS and cost) among pediatric patients. We hypothesized that resource utilization among pediatric patients with a CA comorbidity status would be higher than that among pediatric patients without a CA comorbidity status.

## Methods

### Study Design and Data Source

We conducted a retrospective, cross-sectional study of hospitalized children with CAs from 2013 to 2016 using the child inpatient electronic health record (EHR) database from five tertiary hospitals in Shanghai, China. Among the five hospitals, one is a tertiary pediatric hospital, and the other four are all general tertiary hospitals that have pediatric departments or units of standard size. The EHR data of inpatients with CA disorders in all public health institutions were extracted from the Information Center of Health and Family Planning Commission of Pudong New Area of Shanghai. The Pudong New Area was selected because it represented the average health and economic status of Shanghai (16). In China, due to the large threat of serious medical disputes and deficits in pediatric departments, hospitals are usually not willing to establish pediatric departments unless required by the local government. In this case, pediatric hospitals are always concentrated in developed regions or cities. In our study, the five included tertiary hospitals are located in Shanghai and treat inpatient children from the entire country.

In this study, the population comprised all inpatients aged 3–17 years between 2013 and 2016. Inpatients were classified into the following age groups according to the Chinese Standard for stages of childhood: 3–5 years (preschool age), 6–11 years (school age), and 12–17 years (adolescence). We did not include infants because they were more likely to have single diseases, much longer LOSs and higher hospitalization costs, and the differences between infants and the older children were large, which would lead to large variations. The discharge database for children included the following information: demographic characteristics, billing information, and diagnoses classified by the International Classification of Disease, Ninth Revision (ICD 9), Clinical Modification.

### Variable Measurement

#### Hospital Resource Utilization

Hospital resource utilization included variables of the LOS and the cost of each hospitalization within the CA comorbidity group and the CA-only group. These variables were all continuous. Considering that the variables were not normally distributed, we examined both the mean and median.

#### CA Comorbidity Classifications

A comorbidity was considered present with either a primary or secondary diagnosis of a disease (12). First, we explored whether the hospitalized patient had a CA diagnosis. If there was no CA comorbid diagnosis, then hospitalization was included in the CA-only group. If any CA comorbid disorder was present, the patient was classified into one of two subgroups: (1) another disease as the primary diagnosis and CA as the secondary diagnosis (medical+CA) or (2) CA as the primary diagnosis and another disease as the secondary diagnosis (CA+medical). In the multivariate analysis, the independent variables were the medical+CA, CA+medical, and CA-only groups, and the CA-only group was used as the reference.

#### Demographic and Clinical Characteristics

In the multivariate analysis, the independent variables consisted of demographic and clinical characteristics. The demographic characteristics were sex, age, insurance type, and residence status. Inpatients were classified into one of three age groups: 3–5, 6–11, and 12–17 years. Insurance was classified into three types: governmental (basic insurance for residents), out-of-pocket, and commercial. Among the three types of insurance, the commercial type has the highest reimbursement ratio, followed by the governmental type. We included complex chronic conditions (CCCs) and kinds of surgical operations as clinical characteristics. CCCs were defined according to version 2 of Feudtner's CCCs classification system to reflect whether the inpatient had other chronic diseases and the number of chronic diseases. In this study, the participants were classified into three groups according to the number of chronic diseases: 0, 1, and ≥2 (17). The comorbid condition was based only on primary and secondary diagnoses in our study. Additionally, since surgeries involve greater use of medical services or drugs, the surgical status of the hospitalized individuals was included to reflect the patients' disease condition, and the number of surgical operations was categorized as 0, 1, or ≥2 (18).

### Statistical Analysis

All analyses were performed with SAS, version 9.3 (SAS Institute, Inc., Cary, NC, USA). To assess the distribution of patients with various demographic and clinical characteristics according to the prevalence of CA comorbidity, we used the chi-square test (Cochran–Mantel–Haenszel test). Analysis of variance (ANOVA) was used to explore discrepancies in LOS and cost. By using multivariate linear regression, we estimated multivariate models of the association between the prevalence of the three types of CA status (medical+CA, CA+medical, and CA only) and hospital resource utilization (LOS and cost). In addition, the comorbid diseases of CA patients and the associated hospital resource utilization within the three CA diagnostic groups were further analyzed.

### Ethics Approval

This study was approved by the ethics committees of Tongji University (ref: LL-2016-ZRKX-017). The research presented minimal risk of harm to its subjects, and the data were obtained from hospitalization discharge records that were collected anonymously.

## Results

A total of 7,890 hospitalized children with either a primary or secondary CA diagnosis (13.13%) were included. Of these patients, 542 (6.87%) were categorized into the medical+CA group, 3,480 (44.11%) were in the CA+medical group, and 3,868 (49.02%) were in the CA-only group; 50.98% of the hospitalized patients presented with a comorbid CA condition.

Table 1 shows that among the hospitalized children, demographic characteristics, the prevalence of CCCs, and surgery status varied significantly according to the CA diagnosis status (medical+CA, CA+medical, or CA only). Within the CA+medical group, the proportion of boys was highest (77.93%). More CA comorbidity hospitalizations occurred in the 3–5-year-old age groups of both the medical+CA group (47.97%) and the CA-only group (53.46%). Most of the hospitalized patients (more than 90%) had government insurance in each group. Regarding residence status, we found that the medical+CA (79.70%) and CA-only (78.93%) groups had a higher proportion of nonregistered Shanghai residents. There were more patients in the CA-only group with no CCCs (99.51%) than in the other groups, and only 0.49 and 0.00% had 1 and ≥2 CCCs in this group, respectively. However, in the CA comorbidity groups, more hospitalizations were noted in the CCC = 1 and CCC ≥ 2 subgroups. In addition, the medical+CA group had the highest proportion of hospitalizations without any kind of surgical operations (12.55%), the CA+medical group had the largest proportion of ≥2 kinds of surgical operations (26.29%), and the CA-only group had the highest proportion of one kind of surgical operation (78.46%). The LOS was higher in the medical+CA group, with a mean of 9.35 (10.36) days and a median (IQR) of 7.00 (4.00–11.00) days. Additionally, the average cost was higher in the CA comorbid groups, with average costs of 24.92 and 29.48 thousand RMB in the medical+CA and CA+medical groups, respectively.

**Table 1:** Characteristics of children aged 3–17 years who were hospitalized in tertiary hospitals according to type of comorbid CA (*n*, %).

**Variables**	**Classification**	**CA comorbidity groups**		
		**Medical+CA (*N* = 542)**	**CA+Medical (*N* = 3,480)**	**CA only (*N* = 3,868)**
**DEMOGRAPHICS**				
Sex	Boy	342 (63.10)	2,712 (77.93)	2,345 (60.63)
	Girl	200 (36.90)	768 (22.07)	1,523 (39.37)
	*P-*value		*P < 0.001*	
Age (years)	3–5	260 (47.97)	1,546 (44.43)	2,068 (53.46)
	6–11	203 (37.45)	1,649 (47.39)	1,430 (36.97)
	12–17	79 (14.58)	285 (8.19)	370 (9.57)
	*P*-value		*P < 0.001*	
Insurance type	Government	518 (95.57)	3,413 (98.07)	3,692 (95.45)
	Self-pay	20 (3.69)	63 (1.81)	150 (3.88)
	Commercial	4 (0.74)	4 (0.11)	26 (0.67)
	*P-*value		*P < 0.001*	
Residence status	Nonregistered resident	432 (79.70)	2,417 (69.45)	3,053 (78.93)
	Registered resident	110 (20.30)	1,063 (30.55)	815 (21.07)
	*P-*value		*P < 0.001*	
**CLINICAL STATUS**				
Number of CCCs	0	440 (81.18)	3,145 (90.37)	3,849 (99.51)
	1	80 (14.76)	284 (8.16)	19 (0.49)
	≥2	22 (4.06)	51 (1.47)	0 (0.00)
	*P*-value		*P < 0.001*	
Surgery status	0	68 (12.55)	48 (1.38)	44 (1.14)
	1	355 (65.50)	2,517 (72.33)	3,035 (78.46)
	≥2	119 (21.96)	915 (26.29)	789 (20.40)
	P value		*P < 0.001*	
**HOSPITAL RESOURCE UTILIZATION**				
Length of stay (mean, SD), days		9.35 (10.36)	6.71 (5.34)	6.06 (6.23)
Length of stay (median, IQR), days		7.00 (4.00–11.00)	6.00 (3.00–8.00)	4.00 (3.00–7.00)
	*P*-value		*P < 0.001*	
Cost (mean, SD), thousand RMB		24.92 (30.05)	29.48 (19.38)	19.90 (24.19)
Cost (median, IQR), days		12.52 (5.82–38.55)	33.68 (9.74–42.16)	8.22 (7.02–24.44)
	*P*-value		*P < 0.001*	

*IQR, Interquartile Range*.

*Medical+CA, The primary diagnosis was another disease, and the secondary diagnosis was congenital anomalies*.

*CA+Medical, The primary diagnosis was congenital anomalies and the secondary diagnosis was another disease*.

*CA only, with only congenital anomalies*.

*CCCs, complex chronic conditions*.

Table 2 describes the associations between the type of CA diagnosis (medical+CA, CA+medical, or CA only) and hospital resource utilization (LOS and cost). The results showed that in Model 1, compared with those in the CA-only group, hospitalized patients in the medical+CA group had a significantly longer LOS (β = 2.656, *P* < 0.001), while those in the CA+medical group had a shorter LOS (β = 0.908, *P* < 0.001). Regarding cost, the CA+medical comorbidity group was highly associated with higher cost (β = 7.222, *P* < 0.001), and the medical+CA group was also associated with a higher cost than the CA-only group (β = 4.217, *P* < 0.001).

**Table 2:** Multivariate linear regression of hospital resource utilization (LOS and cost).

**Independent Variable**	**Length of stay**			**Cost**		
	**Model 1**			**Model 2**		
	**β**	***T*-value**	***P*-value**	**β**	***T*-value**	***P*-value**
**DEMOGRAPHICS**						
Sex (*ref boy*)	1.038	6.850	<0.001	10.027	19.150	<0.001
**AGE (YEARS) (*****REF 3–5 YEARS*****)**						
6–11	−0.708	−4.860	<0.001	−5.331	−10.590	<0.001
12–17	−0.492	−1.960	0.050	−2.835	−3.260	0.001
**INSURANCE TYPE (*****REF GOVERNMENT*****)**						
Self-pay	−0.482	−1.120	0.263	−14.250	−9.590	<0.001
Commercial	−1.101	−1.050	0.293	−18.544	−5.120	<0.001
Residence status (*ref nonregistered residents*)	−1.718	−10.650	<0.001	−9.095	−16.300	<0.001
**CLINICAL STATUS**						
**NUMBER OF CCCs (*****REF 0*****)**						
1	2.440	7.470	<0.001	0.349	0.310	0.758
≥2	7.962	11.040	<0.001	13.631	5.460	<0.001
**SURGERY STATUS (*****REF 0*****)**						
1	0.444	0.890	0.372	16.409	9.540	<0.001
≥2	1.291	2.510	0.012	19.131	10.760	<0.001
**COMORBID CA (*****REF CA ONLY*****)**						
Medical+CA	2.656	9.250	<0.001	4.217	4.250	<0.001
CA+medical	0.908	4.090	<0.001	7.222	14.070	<0.001
	Adjusted *R*^2^ = 9.791			Adjusted *R*^2^ = 16.979		

*Medical+CA, The primary diagnosis was another disease, and the secondary diagnosis was congenital anomalies*.

*CA+Medical, The primary diagnosis was congenital anomalies and the secondary diagnosis was another disease*.

*CA only, with only congenital anomalies*.

*CCCs, complex chronic conditions*.

In addition, the inpatients' demographics and clinical status revealed specific effects on hospital resource utilization. Girls tended to have a longer LOS (β = 1.038, *P* < 0.001) and higher cost (β = 10.027, *P* < 0.001). Compared with the 3–5-year-old group, the 6–11-year-old group had a significantly longer LOS (β = −0.708, *P* < 0.001) but a lower cost (β = −5.331, *P* < 0.001), and the 12–17-year-old group also had a lower cost than the 3–5-year-old group (β = −2.835, *P* = 0.001). Regarding insurance type, commercial insurance exhibited a considerably stronger negative association with cost (β = −18.544, *P* < 0.001) than government insurance. Regarding residence status, residents had a shorter LOS (β = −1.718, *P* < 0.001) and lower cost (β = −9.095, *P* < 0.001) than the nonregistered residents. Notably, the CCC status of the hospitalized patients was positively associated with both LOS and cost (*P* < 0.001); the greater the number of CCCs was, the greater the resource utilization in terms of LOS and cost would be (*P* < 0.001). Additionally, the statistical analysis showed that hospitalized patients who underwent more than one kind of surgical operation had a longer LOS (β = 1.291, *P* = 0.012), and the costs were significantly higher in the groups with one surgical operation (β = 16.409, *P* < 0.001) and the group with more than one surgical operation (β = 19.131, *P* < 0.001).

The distributions of the comorbidities in the CA groups and their corresponding hospital resource utilization among the three CA diagnostic groups are shown in Table 3. The top three CA disease types in the three groups were cardiovascular, genitourinary and musculoskeletal diseases. In the medical+CA group, most of the hospitalizations were associated with cardiovascular disease+CA (65.49%), followed by musculoskeletal disease+CA (11.97%) and genitourinary disease+CA (9.23%). The cardiovascular disease+CA hospitalization group had the highest mean cost (29.04 thousand RMB), its mean LOS was 9.91 days, and the median LOS was 7.00 days. In the CA+medical group, CA+cardiovascular diseases were associated with the highest cost (mean = 41.38 thousand RMB, median = 41.32 thousand RMB); however, the associated LOS was short compared with those of other CA+medical subgroups.

**Table 3:** Frequencies of CA comorbid diseases among the CA comorbidity groups.

**Disease system**	**N**	**%**	**Length of stay (days)**				**Cost (thousand RMB)**			
			**Mean**	**SD**	**Median**	**IQR**	**Mean**	**SD**	**Median**	**IQR**
**Group 1: Medical+CA**										
Cardiovascular	355	65.49	9.91	11.68	7.00	4.00–11.00	29.04	33.44	13.72	5.99–46.52
Musculoskeletal	65	11.97	7.58	7.73	6.00	3.00–8.00	24.17	21.47	18.78	9.29–31.47
Genitourinary	50	9.23	7.82	5.63	6.00	4.00–11.00	9.31	6.23	7.81	4.99–12.00
Central nervous	31	5.73	9.32	7.34	9.00	3.00–14.00	16.05	18.02	7.89	3.41–26.14
Gastrointestinal	13	2.39	12.92	7.02	11.00	8.00–18.00	20.81	16.13	19.49	6.25–26.41
Respiratory	11	2.03	6.45	3.91	6.00	3.00–8.00	7.42	3.70	7.21	4.58–10.65
Ears	7	1.29	7.71	12.98	3.00	2.00–5.00	27.95	59.95	7.29	1.76–8.37
Miscellaneous	5	0.92	8.20	5.26	11.00	3.00–12.00	7.27	6.22	5.57	4.21–7.88
Face and neck	3	0.55	7.67	5.51	8.00	2.00–13.00	19.11	12.40	20.78	5.96–30.60
Eyes	2	0.36	10.50	10.61	10.50	3.00–18.00	10.67	3.93	10.67	7.89–13.45
**Group 2: CA+medical**										
Genitourinary	1,918	55.12	4.29	3.41	3.00	3.00–4.00	7.74	3.25	7.29	6.76–7.96
Cardiovascular	1,130	32.48	9.09	8.66	7.00	4.00–10.00	41.38	31.24	41.32	14.33–53.93
Musculoskeletal	298	8.58	4.60	3.14	4.00	3.00–6.00	17.54	15.33	12.79	9.00–20.42
Central nervous	43	1.24	11.21	5.59	13.00	5.00–15.00	29.23	18.63	36.99	6.26–44.12
Gastrointestinal	39	1.12	11.18	7.64	9.00	7.00–15.00	16.24	13.31	13.96	6.28–22.47
Face and neck	21	0.61	4.90	3.03	3.00	2.00–7.00	8.91	5.44	6.62	5.68–13.33
Miscellaneous	18	0.52	6.89	8.29	4.00	3.00–8.00	9.95	11.80	6.91	5.39–9.35
Respiratory	7	0.2	9.86	11.07	6.00	2.00–15.00	14.72	10.31	15.33	5.94–24.23
Eyes	5	0.14	2.20	0.45	2.00	2.00–2.00	8.43	0.93	8.37	7.93–8.54
Ears	1	0.03	3.00	−−	3.00	3.00–3.00	6.30	−−	6.30	6.30–6.30

*Because only one hospitalization was noted in this group, the SD did not exist*.

## Discussion

This study shows that the high occurrence of CA comorbidities reflects the severity of CAs in China to a large extent (7). Although perinatal testing and diagnostic tests (e.g., amniocentesis and chorionic villus sampling) have long been offered to the public (5), insufficient prenatal care knowledge regarding drug use and nutrition (19, 20), as well as new threats from environmental pollution (21, 22), have led to a relatively high prevalence of CAs among children in China. For instance, studies have revealed that in developing countries, including China, exposure of women to air pollutants such as sulfur dioxide, nitrogen dioxide, ozone, and particulate matter (PM) during pregnancy increases the risk of CHDs and other birth defects in infants (22, 23). As revealed in other studies, the high prevalence of CAs has an adverse impact on hospital resource utilization (11, 12). In our study, we found that the presence of a comorbid CA condition had a significant influence on hospital resource utilization among children. This can be explained by the combined CA diagnosis in children; those hospitalized for medical diseases may need more auxiliary equipment and physician inspection. The results showed that hospitalized patients in the comorbid CA+medical group are associated with a higher cost than patients in the medical+CA group. As shown in Table 3, most of the patients in these groups had genitourinary and cardiovascular diseases; however, among the frequently occurring cardiovascular, musculoskeletal, and genitourinary diseases, the CA+medical group was associated with a higher cost. The medical+CA group had a longer LOS than the CA+medical group, which may be explained by the fact that the LOS associated with cardiovascular, musculoskeletal and genitourinary diseases, the top three comorbid diseases, was longer in the medical+CA group than in the CA+medical group. Overall, in each group, the higher LOS and cost of these diseases can be explained by the fact that they are associated with worse hospital outcomes due to pain and other symptoms, greater challenges and risks, and more care coordination and management needs (12, 14, 24). Regarding the highly occurring comorbidities involving cardiovascular diseases, the LOS was longer, and the cost was higher, indicating the seriousness of these comorbidities and that more medical efforts are needed with these diseases to improve care coordination for these patients.

In addition, the statistical analysis revealed some significant effects of demographics on hospital resource utilization. Boys seemed to have a shorter LOS and lower cost, which is not consistent with Cui's (13) study on the discharge sample from Beijing general hospitals in China in which there were no significant sex differences. This may be explained in part by the fact that the proportions of boys and girls were different from those in other studies. Additionally, the younger group (3–5 years) had a shorter LOS than the 6–11- and 12–17-year-old groups, which is not in accordance with Cui's (13) study showing that those in the 5–17-year-old group had a shorter LOS. This difference may be a result of the different comorbid disease statuses and compositions in various studies because in our study; while we focused on all CA diseases, other studies analyzed only certain kinds of CA diseases and did not consider CA comorbid status. The statistical analysis showed that compared with the hospitalized patients in the self-pay and commercial insurance groups, hospitalized patients who had government insurance were likely to have higher costs. Currently, strict restrictions are imposed on the inpatient LOS for individuals with government insurance; however, the cost is not restricted according to the rules. In this study, children with chronic disease conditions, especially those with more than one, were more likely to have a longer LOS and higher cost, consistent with published findings that children with chronic diseases tended to use long-term and more ancillary services or drugs (25, 26). The nonregistered Shanghai residents tended to utilize more hospital resources, mainly because they could not be cured by their local hospitals and had to be treated in tertiary hospitals in developed regions such as Shanghai for more serious conditions (27). In addition, the need for multiple kinds of surgeries was related to a longer LOS and higher cost, which was in accordance with their worse conditions.

The above findings have several potential implications for proper hospital resource utilization. Currently, pediatric clinicians are extensively lacking, even in large-scale hospitals in China. In China, much still remains to be learned on the mechanisms driving the differences in outcomes between patients with and without CA comorbidities, and future research is necessary to understand whether additional resource utilization is beneficial for pediatric patients.

The current study had several limitations. First, the proportion of hospitalized patients with CA and comorbidities was relatively high, mainly because the included hospitals were tertiary hospitals that usually attracted patients with more severe CAs. Second, it was difficult to obtain the inpatients' long-term medical history. Thus, limitations inherent to administrative data may result in the underestimation of the influence of CA conditions on resource utilization. Third, other confounding factors may not have been available in the hospitalization data, including the severity of patients' physical health condition, which could also account for the relationship between health conditions and hospital resource utilization.

## Conclusion

In conclusion, a high prevalence of comorbid CA conditions was observed among pediatric inpatients in the sampled tertiary hospitals in China. The high prevalence of comorbid CAs, especially among pediatric inpatients with primary medical and comorbid CA conditions, merits great attention. The most frequent CA diseases, such as comorbid cardiovascular, musculoskeletal and genitourinary diseases, have a great impact on hospital resource utilization and should receive substantial attention.

## Data Availability Statement

Publicly available datasets were analyzed in this study. This data can be found here: The datasets used and/or analyzed during the current study are available from the corresponding author on reasonable request.

## Ethics Statement

This study was approved by the ethics committees of Tongji University (ref: LL-2016-ZRKX-017). The research presented minimal risk of harm to its subjects, and the data were obtained from hospitalization discharge records that were collected anonymously.

## Author Contributions

Conceived and designed the study: JS, WY, RL, and ZW. Analyzed the data: HJ, ZY, and LL. Contributed reagents, materials and analysis tools: LG, RY, NC, and QL. Wrote the paper: JS, WF, and ZW. Revised the paper: JS and NC. All authors contributed to the article and approved the submitted version.

## Conflict of Interest

The authors declare that the research was conducted in the absence of any commercial or financial relationships that could be construed as a potential conflict of interest.

## Abbreviations

CAs: Congenital anomalies
LOS: Length of stay
CHD: Congenital heart disease
CCCs: Complex chronic conditions
ICD 9: International Classification of Disease, Ninth Revision.
